# Extracorporeal shockwaves therapy for finger stenosing tenosynovitis: a systematic review and meta-analysis

**DOI:** 10.3389/fphys.2026.1714817

**Published:** 2026-02-05

**Authors:** Liyue Zhang, Yuan Luo, Li Chen, Xiong Zhang, Jinli Chen

**Affiliations:** 1 The First People’s Hospital of Neijiang City, Neijiang, Sichuan, China; 2 Southwest Medical University of China, Luzhou, China

**Keywords:** De quervain disease, extracorporeal shockwave therapy, functional outcomes, meta-analysis, pain, systematic review

## Abstract

**Background:**

Stenosing tenosynovitis is a common chronic tendon disease that seriously affects daily life and working ability. However, its treatment is very challenging and requires more effective treatment methods. A large number of clinical studies have shown that extracorporeal shock wave therapy (ESWT) may relieve the symptoms of stenosing tenosynovitis, but there are no published reviews or meta-analyses specifically and comprehensively evaluating its efficacy for this disease. Therefore, through conducting a meta-analysis, this study systematically evaluated the therapeutic effect of ESWT on stenosing tenosynovitis, aiming to provide evidence-based medical evidence for clinical decision-making.

**Methods:**

A literature search was conducted in PubMed, Embase, Web of Science, Cochrane Library, Wanfang, CNKI, and VIP databases. Randomized controlled trials (RCTS) on shock wave therapy for stenosing tenosynovitis from database establishment to June 2025. The limited languages are Chinese and English. The search terms include: “Extracorporeal shockwave therapy,” shock wave therapy, “HIFU therapy, “De quervain stenosing tenosynovitis,” Trigger Digits, “finger Snapping”. The extracted inclusion indicators included the pain score VAS or NRS, the QDASH Disability questionnaire (Quick Disabilities of the Arm, Shoulder, and Hand questionnaire), and the cooney wrist joint function score. After extracting the data, meta-analysis was conducted through Review Manager5.3 software and Stata17.0 software.

**Results:**

Twelve studies were included, all of which were randomized controlled studies. The results showed that the experimental group was significantly superior to the control group in terms of pain relief, with a total effect size of −1.32 (95% CI: −1.85, −0.79), which was highly statistically significant (Z = 4.89, P < 0.00001). The QDASH score of the shock wave group was superior to that of the control group, and there was no statistically significant difference (MD = −6.14, 95% CI [−14.00, 1.72], P = 0.13, I^2^ = 78%). The Cooney score showed that there was a significant difference between the shock wave group and the control group (MD = 13.84, 95% CI [5.04, 22.64], P = 0.002, I^2^ = 95%); The evaluation of clinical efficacy showed that there were significant differences between the shock wave group and the control group (RR = 5.44, 95% CI [2.99, 9.90], P < 0.00001, I^2^ = 49%).

**Conclusion:**

The results of this meta-analysis show that ESWT has a positive effect on symptom improvement in patients with stenosing tenosynovitis, but there is currently a lack of sufficient and high-quality systematic data to support it. In the future, more rigorous and well-designed clinical studies with adequate sample sizes are needed to comprehensively verify the safety and efficacy of ESWT in treating stenotic tenosynovitis.

## 1 Introduction

Stenosing tenosynovitis presents with wrist pain and tenderness at the first dorsal compartment, often involving the abductor pollicis longus (APL) and extensor pollicis brevis (EPB) tendons due to overuse or repetitive activities (Sun et al., 2019). Pain symptoms are often accompanied by thumb abduction, hand grasping movements, and pain with ulnar deviation and other activities (Awan et al., 2017). In histopathologically related studies, tendon sheath thickening and mucoid degeneration have been consistently identified as key pathological features (Stahl et al., 2013). Epidemiological studies have found that the lifetime risk of trigger finger is 2.6%. Women are more likely to be affected than men, especially housewives and physically active individuals (Sun et al., 2024). With the use of various smart devices and the popularization of the Internet, especially mobile phones and computers, the incidence of this disease shows a trend of increasing year by year (Wei et al., 2022). Although such diseases are common and frequently-occurring in clinical practice, because they can cause serious inconvenience to patients’ lives and work, and finger pain symptoms tend to recur after treatment, the treatment methods for such diseases are still one of the key research focuses at present (Suwannaphisit and Chuaychoosakoon, 2022; Challoumas et al., 2023). At present, the clinical treatment of stenosing tenosynovitis mainly includes the following two methods: conservative treatment and surgical treatment. Among them, conservative therapies include splint fixation, non-steroidal anti-inflammatory drugs (NSAIDs), corticosteroid injection, physical therapy, etc. Surgical treatments mainly include direct open surgery, percutaneous release, percutaneous needle release and arthroscopic decompression (Rowland et al., 2015). However, approximately 20%–30% of patients respond poorly to traditional treatments, and hormone injections may lead to complications such as skin pigmentation, subcutaneous tissue atrophy, and fluctuations in blood sugar (De La Barra Ortiz et al., 2025). Surgical intervention is accompanied by trauma risks, postoperative adhesions and economic burden problems (Chung et al., 2022). In recent years, ESWT has gradually been applied in the treatment of stenosing tenosynovitis due to its advantages such as non-invasiveness, convenient operation and few complications, and has become the focus of clinical attention. ESWT is a non-pharmacological approach that utilizes high-energy sound waves. The fESWT is characterized by a field of sound waves that become focused and converge at a specific depth within the human body, with biological effects involving the local cells and tissues (Pesaresi et al., 2025). Its efficacy in treating musculoskeletal diseases such as plantar fasciitis, lateral epicondylitis, calcific tendinitis, nonunion of bones, or delayed union of bones has been recognized (Thomson et al., 2005). ESWT generates mechanical transduction effects to reduce the concentration of pro-inflammatory factors, activates downstream inhibitory systems, and promotes related intracellular chemical reactions and protein synthesis (Wu et al., 2021; Chen et al., 2022). Mechanical stimulation can reduce the expression of matrix metalloproteinases and interleukins related to tendinitis (Chen et al., 2008). ESWT can also increase the material conversion rate of extracellular matrix and induce new blood vessel formation, thereby promoting collagen synthesis for tendon tissue repair (Xiong et al., 2024). Therefore, ESWT has significant potential as it can accelerate inflammation to relieve pain and improve function. However, although many clinical trials have explored the therapeutic effect of ESWT on stenosing tenosynovitis, there are significant differences in the research conclusions. In recent years, many randomized controlled trials (RCTS) on shock wave therapy for stenosing tenosynovitis have been published. Different studies vary significantly in aspects such as sample selection, treatment cycle and efficacy evaluation criteria, and their efficacy results are uneven. In view of the limitations of the existing studies, this article systematically integrates the evidence from randomized controlled trials through meta-analysis to objectively and comprehensively evaluate the clinical efficacy of ESWT for stenosing tenosynovitis, providing a scientific basis for clinical decision-making.

ESWT is a non-pharmacological method that utilizes high-energy sound waves. It achieves anti-inflammatory effects, promotes chemical reactions and tissue repair through mechanical transduction. It has been proven to be effective in treating various musculoskeletal disorders such as plantar fasciitis (Thomson et al., 2005; Wu et al., 2021; Chen et al., 2022; Chen et al., 2008; Xiong et al., 2024). In recent years, many randomized controlled trials (RCTS) on shock wave therapy for stenosing tenosynovitis have been published. Different studies vary significantly in aspects such as sample selection, treatment cycle and efficacy evaluation criteria, and their efficacy results are uneven. In view of the limitations of the existing studies, this article systematically integrates the evidence from randomized controlled trials through meta-analysis to objectively and comprehensively evaluate the clinical efficacy of ESWT for stenosing tenosynovitis, providing a scientific basis for clinical decision-making.

## 2 Data and methods

This meta-analysis fully complies with the Preferred Reporting Items for Systematic Reviews and Meta-analysis (PRISMA) guidelines.

### 2.1 Inclusion criteria for literature

1. Research type: It is a published randomized controlled trial of ESWT for stenosing tenosynovitis.
2. Research subjects: Patients clearly diagnosed with stenosing tenosynovitis. Narrowing of the tendon sheath (radial tuberosity type, flexor tendon type) is diagnosed based on a history of repetitive activities, typical symptoms (local pain accompanied by a positive Finkelstein test or finger snapping and tenderness), and exclusion of other pathologies through imaging. The diagnostic criteria must comply with clinical guidelines, with no restrictions on age, gender, disease duration, etc.
3. Intervention measures: The experimental group received ESWT, including different types (such as focused and divergent), energy density, treatment frequency, treatment cycle, etc. The control group received other intervention measures, such as conservative treatment (corticosteroid injection, indomethacin ointment treatment, physical therapy), placebo (pseudo-shock wave therapy), or blank control group.
4. Outcome indicators: visual analogue scale (VAS) for pain or numerical rating scale (NRS); Quick Disabilities of the Arm, Shoulder and Hand (abbreviated as Quick DASH) Cooney Wrist joint Function score and effectiveness rate.

### 2.2 Literature exclusion criteria

1. Repeated publication of research;
2. Non-randomized controlled studies (such as case reports, reviews, animal experiments, meta-analyses, dissertations, cohort, case-control, etc.);
3. The intervention measures are inconsistent;
4. Literature for which the full text cannot be obtained and data cannot be supplemented through other channels;
5. The diagnostic criteria are not clear;
6. No outcome measures in the inclusion criteria were reported.

### 2.3 Literature retrieval strategy

This study adopted a systematic search strategy. Two researchers independently searched for literature in PubMed, Embase, web of science, Cochrane Library, Wanfang Database, China National Knowledge Infrastructure (CNKI), and VIP Database according to the pre-established criteria. The search time limit for all is from the establishment of the database to June 2025. Chinese search terms include “shock wave”, “extracorporeal shock wave”, “stenosing tenosynovitis of the radial styloid process”, “tenosynovitis”, “trigger finger”, “snapping finger”, etc. The English search terms include “Extracorporeal shockwave therapy”, “De Quervain Disease”, “De Quervain Stenosing Tenosynovitis”, and “Trigger Finger” Disorder”, “Snapping Finger”, “trigger Thumbs”, etc. All search results were exported in EndNote format and checked by a third researcher to ensure the completeness and accuracy of the search. Take PubMed search as an example, as shown in Table 1.

**TABLE 1:** Pubmed search process.

Number	Query	Search details	Results
#7	#3 AND #6	“De Quervain Disease”[MeSH Terms] OR ((((((((((((((((((((((((((((Disease, De Quervain)) OR (De Quervain's Disease)) OR (De Quervains Disease)) OR (De Quervain Stenosing Tenosynovitis)) OR (De Quervain Stenosing Tenosynovitis)) OR (extensor pollicis brevis)) OR (Quervain's disease)) OR (de Quervain tenosynovitis)) OR (Stenosing tenosynovitis)) OR (Trigger Finger Disorder)) OR (Flexor Tendon Entrapment)) OR (Entrapment, Flexor Tendon)) OR (Entrapments, Flexor Tendon)) OR (Flexor Tendon Entrapments)) OR (Tendon Entrapment, Flexor)) OR (Tendon Entrapments, Flexor)) OR (Trigger Digits)) OR (Digits, Trigger)) OR (Digit, Trigger)) OR (Trigger Digit)) OR (Snapping Finger)) OR (Finger, Snapping)) OR (Fingers, Snapping)) OR (Snapping Fingers)) OR (Trigger Thumb)) OR (Thumbs, Trigger)) OR (Thumb, Trigger)) OR (Trigger Thumbs) AND “Extracorporeal shockwave therapy”[MeSH Terms] OR (((((((((((((Extracorporeal shockwave therapy)) OR (Shockwave Therapies, Extracorporeal)) OR (Shockwave Therapy, Extracorporeal)) OR (Therapy, Extracorporeal Shockwave)) OR (Extracorporeal Shock Wave Therapy)) OR (Shock Wave Therapy)) OR (Shock Wave Therapies)) OR (Therapy, Shock Wave)) OR (Extracorporeal High-Intensity Focused Ultrasound Therapy)) OR (High Intensity Focused Ultrasound Therapy)) OR (HIFU Therapy)) OR (HIFU Therapies)) OR (Therapy, HIFU)	16
#6	#4 OR #5	“Extracorporeal shockwave therapy”[MeSH Terms] OR (((((((((((((Extracorporeal shockwave therapy)) OR (Shockwave Therapies, Extracorporeal)) OR (Shockwave Therapy, Extracorporeal)) OR (Therapy, Extracorporeal Shockwave)) OR (Extracorporeal Shock Wave Therapy)) OR (Shock Wave Therapy)) OR (Shock Wave Therapies)) OR (Therapy, Shock Wave)) OR (Extracorporeal High-Intensity Focused Ultrasound Therapy)) OR (High Intensity Focused Ultrasound Therapy)) OR (HIFU Therapy)) OR (HIFU Therapies)) OR (Therapy, HIFU)	12,739
#5	“Extracorporeal shockwave therapy”(Free word)[All Fields]	(((((((((((((Extracorporeal shockwave therapy)) OR (Shockwave Therapies, Extracorporeal)) OR (Shockwave Therapy, Extracorporeal)) OR (Therapy, Extracorporeal Shockwave)) OR (Extracorporeal Shock Wave Therapy)) OR (Shock Wave Therapy)) OR (Shock Wave Therapies)) OR (Therapy, Shock Wave)) OR (Extracorporeal High-Intensity Focused Ultrasound Therapy)) OR (High Intensity Focused Ultrasound Therapy)) OR (HIFU Therapy)) OR (HIFU Therapies)) OR (Therapy, HIFU)	12,739
#4	“Extracorporeal shockwave therapy”[MeSH Terms]	“Extracorporeal shockwave therapy”[MeSH Terms]	3,596
#3	#1 OR #2	“De Quervain Disease”[MeSH Terms] OR ((((((((((((((((((((((((((((Disease, De Quervain)) OR (De Quervain's Disease)) OR (De Quervains Disease)) OR (De Quervain Stenosing Tenosynovitis)) OR (De Quervain Stenosing Tenosynovitis)) OR (extensor pollicis brevis)) OR (Quervain's disease)) OR (de Quervain tenosynovitis)) OR (Stenosing tenosynovitis)) OR (Trigger Finger Disorder)) OR (Flexor Tendon Entrapment)) OR (Entrapment, Flexor Tendon)) OR (Entrapments, Flexor Tendon)) OR (Flexor Tendon Entrapments)) OR (Tendon Entrapment, Flexor)) OR (Tendon Entrapments, Flexor)) OR (Trigger Digits)) OR (Digits, Trigger)) OR (Digit, Trigger)) OR (Trigger Digit)) OR (Snapping Finger)) OR (Finger, Snapping)) OR (Fingers, Snapping)) OR (Snapping Fingers)) OR (Trigger Thumb)) OR (Thumbs, Trigger)) OR (Thumb, Trigger)) OR (Trigger Thumbs)	3,592
#2	De Quervain Disease(Free word)[All Fields]	((((((((((((((((((((((((((((Disease, De Quervain)) OR (De Quervain's Disease)) OR (De Quervains Disease)) OR (De Quervain Stenosing Tenosynovitis)) OR (De Quervain Stenosing Tenosynovitis)) OR (extensor pollicis brevis)) OR (Quervain's disease)) OR (de Quervain tenosynovitis)) OR (Stenosing tenosynovitis)) OR (Trigger Finger Disorder)) OR (Flexor Tendon Entrapment)) OR (Entrapment, Flexor Tendon)) OR (Entrapments, Flexor Tendon)) OR (Flexor Tendon Entrapments)) OR (Tendon Entrapment, Flexor)) OR (Tendon Entrapments, Flexor)) OR (Trigger Digits)) OR (Digits, Trigger)) OR (Digit, Trigger)) OR (Trigger Digit)) OR (Snapping Finger)) OR (Finger, Snapping)) OR (Fingers, Snapping)) OR (Snapping Fingers)) OR (Trigger Thumb)) OR (Thumbs, Trigger)) OR (Thumb, Trigger)) OR (Trigger Thumbs)	3,592
#1	“De Quervain Disease”[MeSH Terms]	“De Quervain Disease”[MeSH Terms]	327

### 2.4 Literature screening and data extraction

#### 2.4.1 Literature screening

Two researchers independently conducted a step-by-step screening of the literature based on the pre-set inclusion and exclusion criteria. First of all, import all the retrieved literature through the EndNote 21 literature management software and delete the duplicate literature. Subsequently, the titles and abstracts of the literature were preliminarily screened to eliminate studies that obviously did not meet the standards, such as non-clinical studies, inconsistent intervention measures, and incorrect research subjects. For the literature with doubts in the initial screening, a secondary screening was conducted after obtaining the full text, and the research design, intervention plan, outcome indicators and other contents were evaluated in detail. If there are differences between the two researchers, they will discuss and resolve them with the third researcher to ensure the accuracy and consistency of the screening results.

#### 2.4.2 Data extraction

After reading the full text of the literature, two researchers extracted the content based on the pre-designed Excel table, including: 1. Basic information: first author, publication year, sample size, country, and research type; 2. Characteristics of the research subjects: age, gender, disease duration; 3. Intervention measures: Shock wave type, energy density, follow-up time, control group intervention method; 4. Primary outcome: VAS score; Secondary outcome: QuickDASH score, Cooney wrist joint function score, clinical efficacy, etc., 5. Risk of bias assessment after the information extraction is completed, the two researchers conduct cross-reviews. If there are any differences, they re-examine the original literature and reach a consensus through full discussion to ensure the accuracy and completeness of the information extraction.

### 2.5 Literature quality evaluation

Two researchers evaluated the included RCTS according to the bias risk assessment methods recommended by the Cochrane Collaboration. The assessment contents included: the generation of random sequences, allocation concealment, blinding of researchers and research subjects, incomplete data, selective reporting, and other bias sources. The degree of offset risk is classified as “low risk”, “high risk” and “unclear”.

### 2.6 Statistical analysis

The data were processed and analyzed using RevMan5.3 software and Stata17.0 software. For binary variables, relative risk (RR) and 95% confidence interval (CI) were used, while for continuous variables, mean difference (MD) and 95%CI were used. Statistical significance was set at p < 0.05. The heterogeneity test was analyzed by chi-square test and I2. When P ≥ 0.05 and I^2^ ≤ 50%, the heterogeneity was relatively small, and the Fixed effect model was adopted. When P < 0.05 or I^2^ > 50%, the heterogeneity was relatively large. The Random effect model was adopted to further explore the sources of heterogeneity, including subgroup analysis, sensitivity analysis and meta-regression.

## 3 Result

### 3.1 Literature search results

**FIGURE 1:**
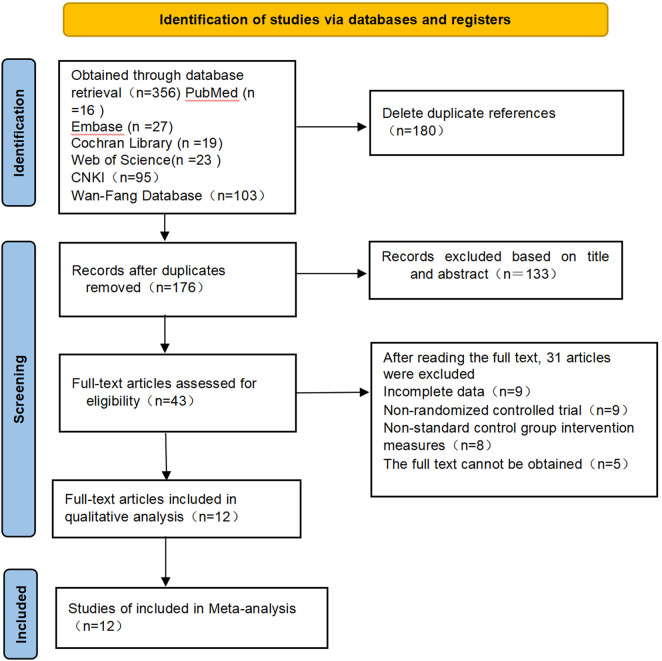
Literature retrieval and screening process.

Through systematic literature retrieval (the retrieval time was up to June 2025), a total of 356 relevant literature were obtained, including English databases: Pubmed (n = 16), Embase (n = 27), Cochran Library (n = 19), Web of Science (n = 23), Chinese databases: China National Knowledge Infrastructure (CNKI, n = 95), Wanfang (n = 103), VIP (n = 73). The NoteExpress 21 software was used to eliminate 180 duplicate literature. The initial screening was conducted by reading the article titles and abstracts, and 133 literature that did not meet the inclusion criteria were excluded. The full text of the remaining 43 literature was read. 31 literature such as incomplete data or no useful outcome measures (n = 9), non-randomized controlled design (n = 9), non-standard control group intervention measures (n = 8), repeated publication or inability to obtain the full text (n = 5) were excluded. Finally, 12 randomized controlled trials (RCTs) were included for meta-analysis. It includes a total of 760 patients. The process of literature retrieval and screening is shown in Figure 1.

### 3.2 Basic characteristics of the included studies

Finally, 12 literature (Yildirim et al., 2016; Chen et al., 2021; Haghighat et al., 2021; Asia Pacific, 2022; Karakuzu Güngör and Güngör, 2025; Jiao et al., 2019; Liu et al., 2015; Dong, 2018; Liu et al., 2014; Ji et al., 2018; Xue et al., 2016; Fu, 2019) published from 2014 to 2025 were included, including 5 in English and 7 in Chinese, with a total of 760 patients, among which 376 were in the experimental group and 384 in the control group. Among all the included studies, shock wave therapy was chosen for stenosing tenosynovitis in the experimental group. In the control group, 2 studies used shock wave placebo, 5 studies used block therapy, 2 studies used conventional physical therapy, and 3 studies used indomethacin ointment for treatment. One article was treated with diclofenac sodium. All studies chose an assessment period of ≥4 weeks or <4 weeks. The basic information of the included literature includes: first author, publication time, treatment method, number of cases, gender proportion, average age, evaluation period, and treatment outcome. However, all 11 studies used VAS scores to assess pain, and 1 study used NRS to assess pain. Five literature evaluated the Quick DASH of Upper Limb Dysfunction Questionnaire score, five literature adopted the Cooney Wrist joint Function score, and six literature evaluated the clinical efficacy. The characteristics of the included studies are shown in Table 2.

**TABLE 2:** Basic characteristics of literature research.

Included studies	Sample size (T/C)	Age (year), mean ± SD	Sex (M:F)	Intervention	Comparison	Period	Outcomes
Karakuzu 2025	29/31	T: 45.4±7.2 C: 42.4±7.8	T: 9/20 C: 7/24	ESWT: Pa1.8 ∼ 1.4bar; Frequency18 ∼ 21Hz; 1000 pulses; Interval:Once/3d	HILT	12wk	①②
Chen 2021	20/20	T: 56.2±8.9 C: 54.8±13.4	T: 5/15 C: 4/16	ESWT: Pa 5.8bar; 1500 puls; Interva: lOnce/7d	Sham Group	12WK	①②
Asia Pacific, 2022	15/15	T: 56.4±11.5 C: 56.4±11.5	T: 2/13 C: 2/13	ESWT: (EFD) 0.03mJ/mm2–0.14mJ/mm2; Frequency 4Hz; 1600pulses; Interval: Once/7d	Steroidal	12WK	①②
Haghighat et al., 2021	13/13	T: 44.61±11.36 C: 48.23±14.45	T: 6/7 C: 4/9	ESWT: Pa 2bar; Frequency15Hz; 1000 pulses; Interval: Once/7d	Sham group	6WK	①②
Yildirim 2016	20/20	T: 55±8 C: 54±9	T: 4/16 C: 3/17	ESWT: Pa 2.1bar; Frequency15Hz; 1000pulses; Interval: Once/7d	Injection	12WK	①②
Jiao et al., 2019	30/30	T: 20-72(median age) C: 22-71(median age)	T: 12/18 C: 15/15	ESWT: Pa 1.0∼1.5bar; Frequency8Hz; 2000 pulses; Interval: Once/4d	Local injection	12WK	①④
Liu 2015	29/27	T: 45.2±6.1 C: 43±6.4	T: 8/21 C: 9/18	ESWT: Pa 1∼2bar; Frequency3Hz; 2000 pulses; Interval: Once/7d	Diclofenac sodium	4WK	①③④
Dong 2018	43/43	T: 44.8±5.6 C: 42.6±6.4	T: 20/23 C: 19/24	ESWT: Pa 2bar; Frequency6∼ 7Hz; 2000pulses; Interval: Once a week	Microwave therapy	3WK	①④
Liu 2014	57/55	T: 42.8±5.1 C: 40.6±6.6	T: 8/49 C: 7/48	ESWT: Pa 1.5∼4.0bar; Frequency8∼12Hz; 1500∼2000 pulses; Interval: Once/5d	Local injection	4WK	③④⑤
Ji et al., 2018	30/30	T: 45.2±6.8 C: 44.8±6.9	T: 9/21 C: 10/20	ESWT: Pa2.0∼ 3.0bar; Frequency8∼12Hz; 2000 pulses; Interval Once/7d	Local injection	4WK	①③④
Xue 2016	40/40	T: 46.4±7.2 C: 45.8±6.8	T: 14/26 C: 12/28	ESWT: Pa2.0∼3.0bar; Frequency8∼12Hz; 2000 pulses; Interval Once/7d	Indomethacin ointment	4WK	①③
Fu 2019	30/30	T: 44.31±2.67 C: 44.42±2.58	T: 10/20 C: 12/18	ESWT: Pa1.6∼2.6bar; Frequency8∼12Hz; 600 pulses	Indomethacin ointment	4WK	①③④

The numbers correspond to the outcome indicators as follows: ① VAS=visual analogue scale1 ② QDASH=Quick Disabilities of the Arm, Shoulder and Hand ③ Cooney Wrist Joint Function Score ④ Response Rate ⑤ NRS=Numerical Rating Scale for Pain; T=test group C=control group; M=Male F=Female.

### 3.3 Risk of bias and quality assessment

**FIGURE 2:**
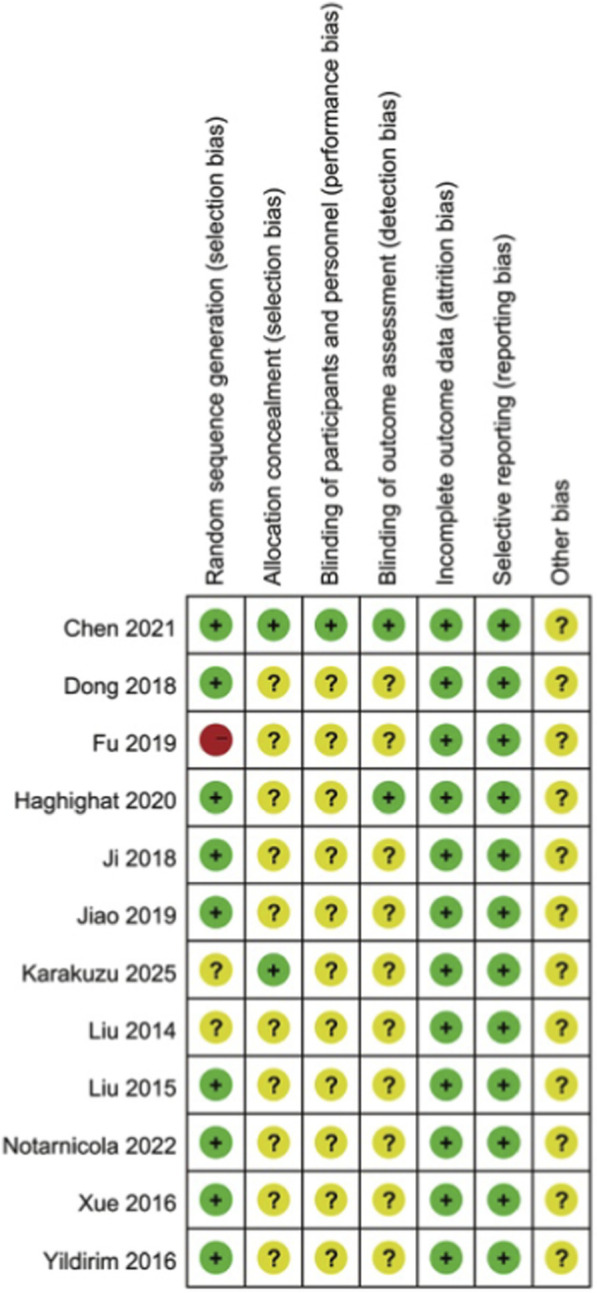
Summary of bias risks.

**FIGURE 3:**
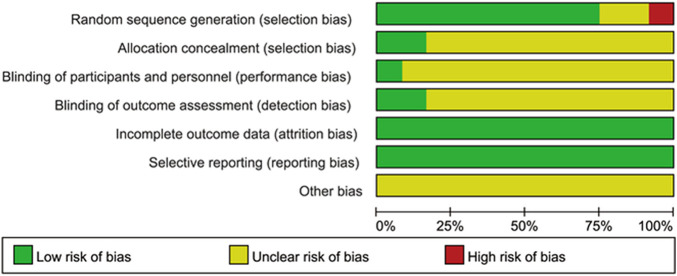
Risk of bias map.

According to the instructions in the Cochrane Handbook of Systematic Reviews of Interventions (Vavken et al., 2009) (Figures 2, 3), randomized controlled trials related to the risk of bias in seven aspects were evaluated. All the studies were randomized controlled trials. The generation of random sequences was either computer-generated or allocated and hidden using sealed envelopes. Among them, 3 literature adopted the random number table method, and 6 literature mentioned the software randomization method. Three literature did not clarify the random method. Regarding the concealment of the allocation scheme, two adopted the sealed envelope method, and the rest were unclear. One literature reported double-blinding of patients, researchers and evaluators, one reported blinding of subjects, and the remaining literature did not mention the blinding situation. Two literature mentioned cases of dropout. The causes of dropout were not related to the intervention effect and were determined to be of low risk. The summary and chart of the risk of bias assessment are shown in Figures 2, 3.

### 3.4 Meta-analysis results

#### 3.4.1 Pain score

##### 3.4.1.1 Subgroup analysis based on the intervention measures of the control group

**FIGURE 4:**
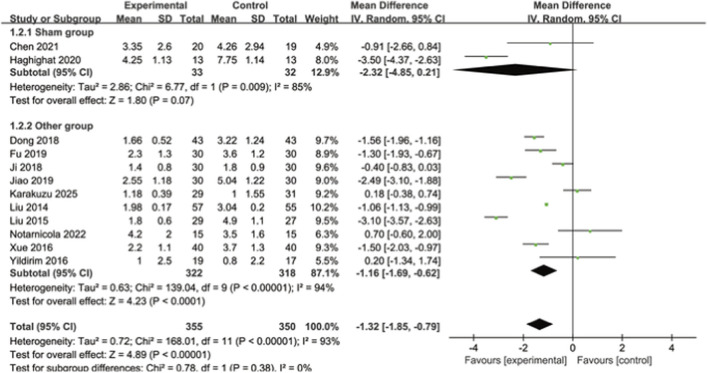
Forest map of subgroup analysis in the VAS control group.

Twelve studies reported pain scores, among which 11 used the VAS score scale and 1 used the NRS scale. A total of 705 patients were included. The smaller these pain score values are, the milder the pain degree of the patients will be. The heterogeneity among the studies was relatively large. The random effects model was selected to analyze the research data. The results showed that the pain score of the experimental group was significantly better than that of the control group, and the difference was statistically significant [MD = −1.32, 95% CI (−1.85, −0.79), I^2^ = 93%, P < 0.00001]. Subgroup analysis was conducted according to the intervention measures of the control group. When the control group was treated with placebo, the results showed that the pain score of the experimental group was lower than that of the control group, and there was no statistically significant difference [MD = −2.32, 95% CI (−4.85, 0.21), I^2^ = 85%, P = 0.07]; When the control group received other treatments, the results showed that the pain score of the experimental group was lower than that of the control group, and the results were statistically different [MD = −1.16, 95% CI (−1.69, −0.62), I^2^ = 94%, P < 0.0001]. It indicates that extracorporeal shock wave can significantly improve pain compared with other treatment measures. As shown in Figure 4.

##### 3.4.1.2 Subgroup analysis by evaluation period

**FIGURE 5:**
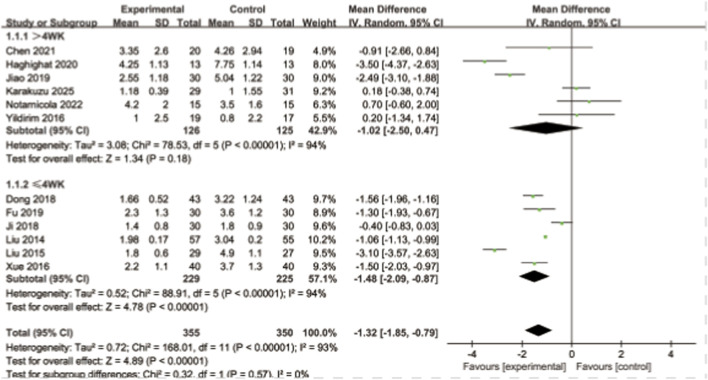
Forest plot of VAS time subgroup analysis.

Twelve studies reported pain scores. A total of 705 patients were included. The smaller these pain score values are, the milder the pain degree of the patients will be. The heterogeneity among the studies was relatively large. Using the random effects model, the results showed that the pain score of the experimental group was significantly lower than that of the control group, and the difference was statistically significant [MD = −1.32, 95% CI (−1.85, −0.79), I^2^ = 93%, P < 0.00001]. Subgroup analysis according to the assessment cycle showed that when the assessment cycle was >4 weeks, the results indicated that the pain score of the experimental group was lower than that of the control group, with no statistically significant difference [MD = −1.02, 95%CI (−2.50, 0.47), I^2^ = 94%, P = 0.18]. When the evaluation period was ≤4 weeks, the results showed that the pain score of the experimental group was lower than that of the control group, and the difference was statistically significant [MD = −1.48, 95% CI (−2.09, −0.87), I^2^ = 94%, P < 0.00001]. It indicates that extracorporeal shock wave can effectively alleviate pain symptoms in the short term, and the long-term pain relief gradually decreases. As shown in Figure 5.

##### 3.4.1.3 Perform subgroup analysis based on the type of shock wave

**FIGURE 6:**
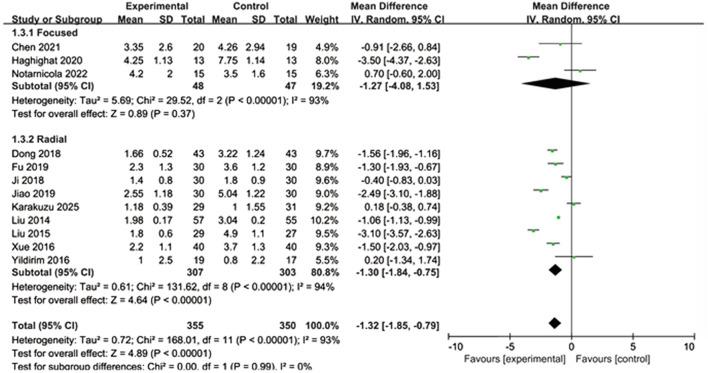
Perform subgroup analysis based on the type of shock wave.

A total of 12 studies were included, involving 705 participants. In the focused shock wave subgroup, the pooled average difference was not statistically significant (MD = −1.27, 95% CI: −4.08 to 1.53, P = 0.37), and there was a high degree of heterogeneity (I^2^ = 93%). In the radiation shock wave subgroup, the experimental group showed statistically significant benefits (MD = −1.30, 95% CI: −1.84 to −0.75, P < 0.00001), although the heterogeneity was still high (I^2^ = 94%). The overall pooled effect of all studies was statistically significant (MD = −1.32, 95% CI: −1.85 to −0.79, P < 0.00001), and the subgroup difference test indicated no significant differences among different shock wave types (P = 0.99, I^2^ = 0%). As shown in Figure 6.

#### 3.4.2 QDASH score

**FIGURE 7:**
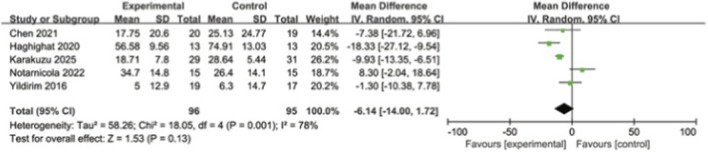
QDASH forest map.

There were 5 literature that adopted the QDASH score in the study, and a total of 91 patients were included. The lower the QDASH score is, the better the recovery of the patient’s upper limb function is indicated. Heterogeneity analysis showed that there was significant heterogeneity among the studies. Therefore, a random effects model was adopted. The results indicated that the QDASH score of the experimental group was significantly higher than that of the control group, with no statistical difference [MD = −6.14, 95% CI (−14.00, 1.72), I^2^ = 78%, P = 0.13), suggesting that extracorporeal shock wave had no significant improvement in upper limb function. As shown in Figure 7.

#### 3.4.3 Cooney score

**FIGURE 8:**
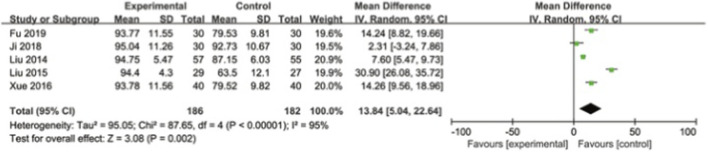
Cooney forest map.

Five studies were included to evaluate the improvement of the Cooney score, with a total of 368 cases. The higher the Cooney score is, the better the wrist joint function of the patient will be. The heterogeneity among the studies was relatively large. A random effects model was used. The results showed that the Cooney score of the experimental group was significantly higher than that of the control group, with a statistically significant difference [MD = 13.84, 95% CI (5.04, 22.64), I^2^ = 95%, P = 0.002]. It indicates that extracorporeal shock wave can significantly improve wrist joint dysfunction, as shown in Figure 8.

#### 3.4.4 Overall clinical effectiveness rate

**FIGURE 9:**
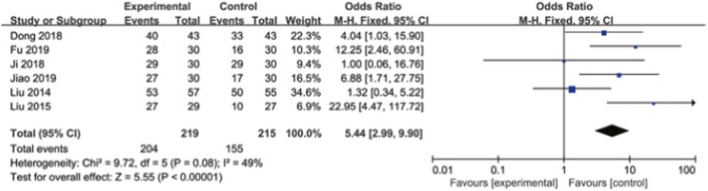
Clinical effects forest plot

Six studies reported the clinical efficacy, involving a total of 434 patients. There was moderate heterogeneity among the studies. A fixed-effect model was used. The results showed that the clinical efficacy of the experimental group was better than that of the control group, with a statistically significant difference [OR = 5.44, 95% CI (2.99, 9.90), I^2^ = 49%, P = 0.008]. The results show that compared with other measures, extracorporeal shock wave can significantly improve the clinical efficacy of stenosing tenosynovitis. As shown in Figure 9.

### 3.5 Multiple regression analysis

Multiple regression analysis revealed that none of the examined variables—country, publication year, time period, intervening measures, or research setting quality—demonstrated a statistically significant association with the effect size. As shown in Table 3.

**TABLE 3:** The results of the multiple regression analysis.

Covariates	Comparision	Coefficient	Standard error	t	P
Country	12	2.159	2.55	0.85	0.429
Publish	12	0.186	2.21	0.08	0.936
Period	12	−0.172	1.95	−0.09	0.932
IM	12	3.354	2.26	1.48	0.189
RSQ	12	4.914	3.64	1.35	0.225

IM=Intervening measure; RSQ=research on setting quality.

### 3.6 Sensitivity analysis

**FIGURE 10:**
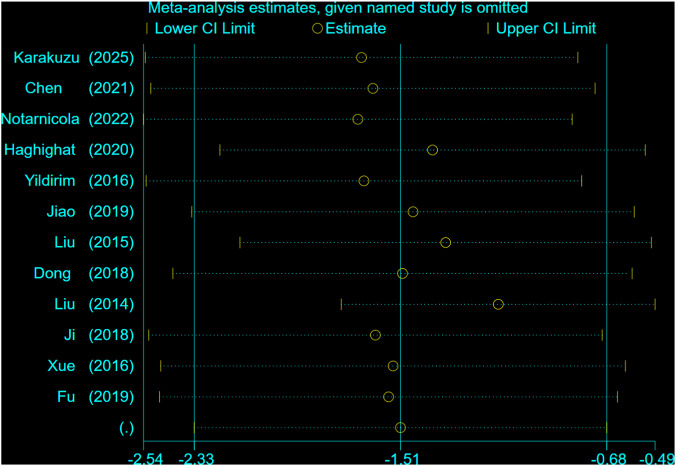
Sensitivity analysis of pain conditions in two groups of patients with stenosing tenosynovitis.

Sensitivity analysis employing the leave-one-out method confirmed the stability of the pooled estimate. Sequential exclusion of individual studies yielded consistently negative 95% confidence intervals, supporting the robustness of the results. No single study significantly altered the magnitude or direction of the pooled effect. As shown in Figure 10.

### 3.7 Publication bias

**FIGURE 11:**
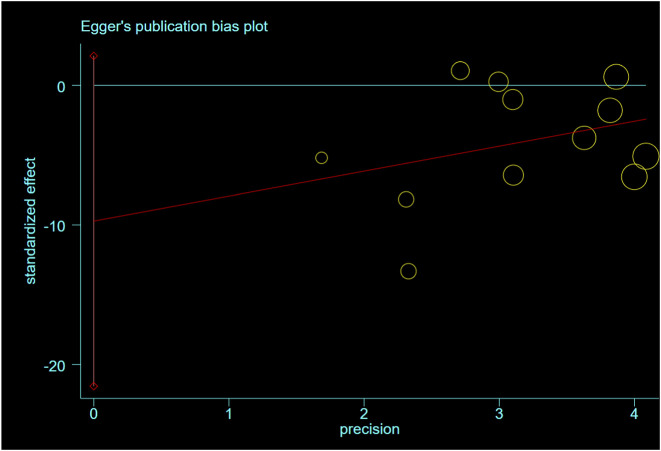
Begg’s funnel plot with pseudo 95% confidence limits.

**FIGURE 12:**
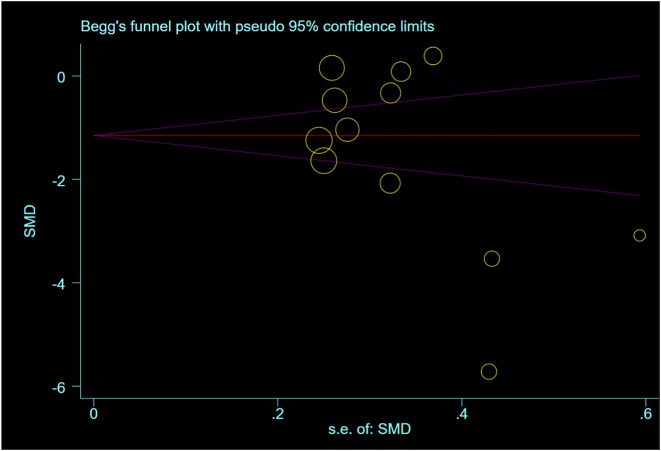
Egger’s publication bias plot.

The Egger test and Begg test were used to assess the bias of the main outcome measure, pain results of tenosynovitis, in rotator cuff injuries. Both tests indicated the absence of significant bias (Egger test: P = 0.703; Begg test: P = 0.584). As shown in Figures 11, 12.

## 4 Discussion

### 4.1 Summary of main results

This study is the first systematic review and meta-analysis based on randomized controlled trials (RCTS), aiming to explore the clinical effect of ESWT on stenosing tenosynovitis. The study focused on indicators such as the Visual Analogue Scale (VAS) for Pain or the Numerical Rating Scale (NRS), the score of the Upper Limb Dysfunction Questionnaire, the Cooney Wrist Joint Function Score, and the effective rate, and conducted an integrated analysis of the data of a total of 760 patients with stenosing tenosynovitis from 12 RCT studies. The subgroup analysis on pain relief results indicated that short-term pain relief was significant (p < 0.0001), while long-term effects gradually decreased (p = 0.18). This might be related to the short-term effect of extracorporeal shock waves. Additionally, when the control group used a placebo (p = 0.07), although there was no significant difference in the treatment effect of extracorporeal shock waves, this near-significant finding might suggest a potential therapeutic effect. Larger-scale and more effective studies might be able to confirm this result. Compared to the placebo, shock waves have a better pain relief effect than other drugs and physical therapies (p < 0.001). This might be due to the placebo effect, or the “instant stimulation” of shock wave therapy, which may trigger the activation of injury repair-related pathways and stimulate local inflammatory responses (therapeutic inflammation, which is beneficial for subsequent repair but temporarily aggravates pain perception), causing patients to perceive pain more significantly than the placebo group in the assessment of pain within a short period (Li et al., 2022). The application of extracorporeal shockwave therapy (ESWT) demonstrated statistically significant and clinically meaningful improvements in both wrist joint functionality and overall therapeutic outcomes. In this Meta-analysis, although the Cooney score was positive, the QDASH score did not show significant improvement. This outcome might be due to the sensitivity and responsiveness. QuickDASH is a general upper limb function assessment scale, while the Cooney score is specifically for the wrist area. For the treatment of narrow tenosynovitis, the Cooney score has a higher sensitivity to changes in the condition. Minor but statistically significant changes in specific clinical indicators may not be captured by a broader functional assessment questionnaire covering the entire arm and shoulder. Although shock waves can promote tissue repair through mechanical stress, their improvement effect on structural changes such as thickening of the tendon sheath and tendon adhesion that have already formed is limited, and it is difficult to significantly improve the functional scores such as grasping and lifting of patients. Tendinosis of the flexor tendons, fibrotic thickening of the finger pulley, and tenosynovitis of the tendon sheath can be considered sonographic patterns with different responses to the shockwaves (Kucera, 1977). Furthermore, the high heterogeneity of treatment parameters (energy density, number of shocks) and baseline characteristics of patients (disease duration, severity of the disease) in the included studies may further weaken the potential therapeutic effect. The results of this analysis indicate that for the treatment of stenosing tenosynovitis with the core goal of improving upper limb function, more high-quality studies are still needed to verify the effectiveness of shock wave therapy.

Modern medical research shows that the etiology of stenosing tenosynovitis is related to chronic strain, mechanical compression, endocrine disorders, anatomical abnormalities, infectious factors and other factors (Vuillemin et al., 2012). Repeated flexion and extension of fingers or wrists leading to excessive friction between tendons and tendon sheaths, long-term holding of hard objects causing local compression, endocrine changes caused by pregnancy or hypothyroidism, and anatomical factors such as congenital abnormal tendon course or tendon sheath stenosis may all induce this disease (Ponomareva et al., 1979; Fakoya et al., 2023). Its pathogenesis is considered to be related to the inflammatory response of tendon sheath tissue, tendon degeneration, imbalance of synovial fluid secretion and biomechanical abnormalities (Hittmair et al., 2012). Local inflammatory medium infiltration leads to thickening and fibrosis of the tendon sheath. Long-term compression of the tendon results in edema and deformation. The reduction of synovial fluid secretion intensifies friction. The abnormal force application pattern of the hand alters the biomechanical balance of the joint, which is a key link in the progression of the disease (Cheng et al., 2013; Liu et al., 2022; Choi et al., 2025). This disease is more common among manual workers, middle-aged and elderly people, and postpartum women. Through conservative treatment (such as medication and physical therapy) or surgical intervention, the symptoms can be relieved. However, conservative treatment is prone to recurrence, surgery has the risks of trauma and complications, and currently there is a lack of specific treatment methods targeting the pathological mechanism (Hittmair et al., 2012; Ferrara et al., 2020). Based on this, shock wave therapy has shown unique advantages and broad prospects in clinical applications due to its characteristics of non-invasiveness, strong targeting and few complications.

In recent years, the clinical research and mechanism exploration of shock wave therapy for stenosing tenosynovitis have been increasingly in-depth and have received extensive attention in the field of rehabilitation medicine (Malliaropoulos et al., 2016). A large number of clinical studies and basic experiments have confirmed that ESWT can not only effectively relieve pain and improve the function of the carpal and finger joints, but also exert therapeutic effects by regulating inflammatory responses and tissue repair mechanisms (Auersperg and Trieb, 2020; Charles et al., 2023). Li et al. - By constructing a rat model of tenosynovitis, it was found that shock wave therapy could activate the PI3K/Akt signaling pathway, promote the proliferation of fibroblasts and the synthesis of type I collagen, and simultaneously reduce the abnormal expression of matrix metalloproteinase-9 (MMP-9) in tendon tissue, thereby improving the structure and function of tendons and tendon sheathing (Chen et al., 2004; 2022; Auersperg and Trieb, 2020). Furthermore, clinical studies have shown that ESWT can significantly enhance patients’ grip strength, reduce the VAS pain score, and improve the Cooney wrist joint function score, confirming its effectiveness in alleviating symptoms and promoting functional recovery (Asia Pacific, 2022).

### 4.2 Limitations

Although this meta-analysis provides important evidence-based medical evidence for ESWT in the treatment of stenosing tenosynovitis, there are still certain limitations. Firstly, there is heterogeneity in the included studies. Different studies have differences in the selection of shock wave types (such as RSWT (Radial Shock Wave Therapy), FSWT (Focused Shock Wave Therapy), etc.), which may lead to different therapeutic effects. Moreover, the diversity of energy parameter Settings (such as energy density, impact times, frequency, etc.) makes it difficult to directly compare and summarize the research results. Furthermore, the control group Settings were also not the same. Some studies used placebo ESWT as the control, while others used drug treatment or other conservative treatment methods as the control, which increased the uncertainty of the results. Secondly, there is a significant lack of long-term follow-up data. Although this is sufficient to demonstrate the short-term efficacy and safety, no conclusion can be drawn regarding the long-term durability of the therapeutic effect after this period. The observed benefits may weaken over time, so re-treatment may be necessary. Due to the lack of long-term follow-up data, a comprehensive assessment of the long-term effect of ESWT cannot be made. Furthermore, there is a risk of publication bias. Since the publication of research results often tends to favor those with positive outcomes, there may be some unpublished negative studies, which may lead to the meta-analysis results overestimating the efficacy of ESWT. Furthermore, the included randomized controlled trial studies also have methodological flaws, such as unclear blinding procedures and unsecured allocation processes. In view of these limitations, the future research directions have clear goals. Firstly, multi-center, large-sample, double-blind randomized controlled trials (RCTS) should be carried out. Multicenter studies can include a broader patient population and reduce the limitations of the sample. A large sample size can improve the stability and reliability of the research results; Double-blind design can effectively reduce bias in the research process and improve the quality of research. By unifying the parameter Settings of ESWT (such as shock wave type, energy density, treatment frequency, etc.) and control protocols, better comparability is achieved among different studies, thereby providing more reliable evidence for the treatment of stenosing tenosheath with ESWT. Secondly, combined with imaging indicators such as ultrasound or MRI, explore the predictive factors of therapeutic effect. Ultrasound and MRI can clearly display the lesion conditions of the tendons, tendon sheaths and other tissue structures in patients with stenosing tenosynovitis, such as the thickness of the tendons, the amount of fluid accumulation in the tendon sheaths, and whether there is calcification, etc. By analyzing the relationship between these imaging indicators and the therapeutic effect of ESWT and seeking the factors that can predict the therapeutic effect, it is helpful to stratify patients before treatment, formulate more precise treatment plans for different patients, and improve the pertinence and effectiveness of treatment.

### 4.3 Future directions

With the continuous deepening of research, the mechanism of ESWT in treating stenosing tenosynovitis will become clearer, and the treatment plan will be more precise and personalized. Combining imaging techniques to explore efficacy predictors and studying the combined application of ESWT with other therapies will further expand the application scope of ESWT in the treatment of stenosing tenosynovitis, improve the therapeutic effect, and bring more benefits to a large number of patients. It is believed that in the near future, ESWT will play a more significant role in the treatment of stenosing tenosynovitis, providing clinicians with more powerful therapeutic weapons and offering patients better medical services.

## 5 Conclusion

In conclusion, the results of this meta-analysis indicate that ESWT is effective in relieving pain and improving function for patients with stenosing tenosynovitis, and it is also safe. However, the current research still has some shortcomings and requires further high-quality studies to be conducted for improvement.

## Data Availability

The original contributions presented in the study are included in the article/supplementary material, further inquiries can be directed to the corresponding author.

## References

[B1] Asia Pacific (2022). Effect of Shock Waves in the Treatment of De Quervain’s Syndrome: a Radomized Perspective Clinical Study. J. Biol. Regul. Homeost. Agents 36. 10.23812/j.biol.regul.homeost.agents.20223604.92

[B2] Auersperg V. Trieb K. (2020). Extracorporeal shock wave therapy: an update. EFORT Open Rev. 5, 584–592. 10.1302/2058-5241.5.190067 33204500 PMC7608508

[B3] Awan W. A. Babur M. N. Masood T. (2017). Effectiveness of therapeutic ultrasound with or without thumb spica splint in the management of De Quervain’s disease. BMR 30, 691–697. 10.3233/BMR-160591 28035912

[B4] Challoumas D. Ramasubbu R. Rooney E. Seymour-Jackson E. Putti A. Millar N. L. (2023). Management of de Quervain Tenosynovitis: a Systematic Review and Network Meta-Analysis. JAMA Netw. Open 6, e2337001. 10.1001/jamanetworkopen.2023.37001 37889490 PMC10611995

[B5] Charles R. Fang L. Zhu R. Wang J. (2023). The effectiveness of shockwave therapy on patellar tendinopathy, achilles tendinopathy, and plantar fasciitis: a systematic review and meta-analysis. Front. Immunol. 14, 1193835. 10.3389/fimmu.2023.1193835 37662911 PMC10468604

[B6] Chen Y. Wang C. Yang K. D. Kuo Y. Huang H. Huang Y. (2004). Extracorporeal shock waves promote healing of collagenase‐induced achilles tendinitis and increase TGF‐β1 and IGF‐I expression. J. Orthop. Res. 22, 854–861. 10.1016/j.orthres.2003.10.013 15183445

[B7] Chen Y.-J. Huang C.-H. Lee I.-C. Lee Y.-T. Chen M.-H. Young T.-H. (2008). Effects of cyclic mechanical stretching on the mRNA expression of Tendon/ligament-related and osteoblast-specific genes in human mesenchymal stem cells. Connect. Tissue Res. 49, 7–14. 10.1080/03008200701818561 18293173

[B8] Chen Y.-P. Lin C.-Y. Kuo Y.-J. Lee O. K.-S. (2021). Extracorporeal shockwave therapy in the treatment of trigger finger: a randomized controlled study. Archives Phys. Med. Rehabilitation 102, 2083–2090.e1. 10.1016/j.apmr.2021.04.015 34029555

[B9] Chen K.-T. Chen Y.-P. Kuo Y.-J. Chiang M.-H. (2022). Extracorporeal shock wave therapy provides limited therapeutic effects on carpal tunnel syndrome: a systematic review and meta-analysis. Medicina 58, 677. 10.3390/medicina58050677 35630095 PMC9144370

[B10] Cheng J. Carr C. Wong C. Sharma A. Mahfouz M. Komistek R. (2013). Altered spinal motion in low back pain associated with lumbar strain and spondylosis. Evidence-Based Spine-Care J. 04, 006–012. 10.1055/s-0033-1341640 24436694 PMC3699246

[B11] Choi Y. K. Sit R. W.-S. Wang B. Cheuk C. Lee M. K. Leung K. W. M. (2025). Clinical effectiveness of finger gliding exercise for patients with trigger fingers receiving steroid injection: a randomized clinical trial. Sci. Rep. 15, 5141. 10.1038/s41598-025-89436-9 39934311 PMC11814069

[B12] Chung K. Y. Ho G. Novak C. B. Baltzer H. L. (2022). Aromatase inhibitor–induced carpal tunnel syndrome and stenosing tenosynovitis: a systematic review. Plastic and Reconstr. Surg. 149, 445e–452e. 10.1097/PRS.0000000000008835 35196681

[B43] De La Barra Ortiz H. A. Parizotto N. A. Chamorro Lange C. Liebano R. E. (2025). Effects of high-intensity laser therapy in patients with De Quervain’s tenosynovitis: a systematic review and meta-analysis. J. Hand Ther., S0894113024001455. 10.1016/j.jht.2024.10.001 39814632

[B13] Dong J.-C. (2018). Efficacy observation of radial shock wave therapy for stenosing tenosynovitis. Massage and Rehabilitation Med. 6, 31–33. 10.19787/j.issn.1008-1879.2018.06.014

[B14] Fakoya A. O. Tarzian M. Sabater E. L. Burgos D. M. Maldonado Marty G. I. (2023). De Quervain’s Disease: a Discourse on Etiology, Diagnosis, and Treatment. Cureus 15, e38079. 10.7759/cureus.38079 37252462 PMC10208847

[B15] Ferrara P. E. Codazza S. Cerulli S. Maccauro G. Ferriero G. Ronconi G. (2020). Physical modalities for the conservative treatment of wrist and hand’s tenosynovitis: a systematic review. Seminars Arthritis Rheumatism 50, 1280–1290. 10.1016/j.semarthrit.2020.08.006 33065423

[B16] Fu J.-D. (2019). Analysis of the clinical efficacy of extracorporeal shock wave therapy for stenosing tenosynovitis of the styloid process of the radius. World Compos. Med. 1, 102–104. 10.11966/j.issn.2095-994X.2019.05.01.34

[B18] Haghighat S. Vahdatpour B. Ataei E. (2021). The effect of extracorporeal shockwave therapy on de Quervain Tenosynovitis; a clinical trial. Shiraz E-Med J. 22. 10.5812/semj.106559

[B19] Hittmair K. M. Groessl V. Mayrhofer E. (2012). Radiographic and ultrasonographic diagnosis of stenosing tenosynovitis of the abductor pollicis longus muscle in dogs. Vet. Radiol. Ultrasound 53, 135–140. 10.1111/j.1740-8261.2011.01886.x 22118578

[B45] Ji L-T. Wu Y-H. Han W-D. (2018). Clinical efficacy observation of three methods in the treatment of stenosing tenosynovitis of the styloid process of the radius. Biomed. Orthop. Mater. Clin. Res. 1, 71–73. 10.3969/j.issn.1672-5972.2018.01.018

[B20] Jiao P.-Z. Feng H. Zhao Z.-J. Yu W.-H. Pu Q.-K. (2019). Divergent shock wave therapy for stenosing of the flexor tendons of the thumb clinical observation of tenosynovitis. Chinesse Convalescent Medicine 28, 156–158. 10.13517/j.cnki.ccm.2019.02.019

[B21] Karakuzu Güngör Z. Güngör E. (2025). The comparison of the efficacy of extracorporeal shockwave therapy and high-intensity laser therapy in the treatment of de Quervain tenosynovitis. Turk J. Phys. Med. Rehab 71, 28–36. 10.5606/tftrd.2024.14671 40270626 PMC12012914

[B22] Kucera W. R. (1977). What the nurse anesthetist should know about the law’s view of informed consent. J. Am. Assoc. Nurse Anesth. 45, 309–311. 11664832

[B23] Li C. Xiao Z. Chen L. Pan S. (2022). Efficacy and safety of extracorporeal shock wave on low back pain: a systematic review and meta-analysis. Medicine 101, e32053. 10.1097/MD.0000000000032053 36595991 PMC9803516

[B24] Liu L.-M. Shang H.-S. Zheng Y.-Y. (2014). Efficacy observation of radial shock wave therapy for stenosing tenosynovitis of the styloid process of the radius. China Rehabil. 6, 439–441. 10.3870/zgkf.2014.06.013

[B25] Liu Y. Wu K. Liu S.-T. Zhao Z. Xing G.-Y. (2015). Efficacy Observation of Radial Extracorporeal Shock Wave Therapy for De Quervain's Tenosynovitis. Chin. J. Curr. Adv. Med. 7, 18–20.

[B26] Liu H. Xing Y. Wu Y. (2022). Effect of wii fit exercise with balance and lower limb muscle strength in older adults: a meta-analysis. Front. Med. 9, 812570. 10.3389/fmed.2022.812570 35602499 PMC9120538

[B27] Malliaropoulos N. Jury R. Pyne D. Padhiar N. Turner J. Vasileios K. (2016). Radial extracorporeal shockwave therapy for the treatment of finger tenosynovitis (Trigger digit). OAJSM 7, 143–151. 10.2147/OAJSM.S108126 27843364 PMC5098764

[B28] Pesaresi A. Ricci V. Farì G. Donati D. Chang K. Gervasoni F. (2025). Focal extracorporeal shockwave therapy in shoulder calcific tendinopathy: a retrospective observational study of sonographic prognostic factors. PMRJ, 13454. 10.1002/pmrj.13454 40767460 PMC12903625

[B29] Ponomareva V. L. Burykina L. N. Vasil’eva L. A. Aĭzina N. L. Veselovskaia K. A. (1979). Remote sequelae of the effect of the dust-radiation factor combined with chronic gamma-irradiation on the body of rats. Gig. Tr. Prof. Zabol., 34–37. 544324

[B30] Rowland P. Phelan N. Gardiner S. Linton K. N. Galvin R. (2015). The Effectiveness of Corticosteroid Injection for De Quervain’s Stenosing Tenosynovitis (DQST): a Systematic Review and Meta-Analysis. TOORTHJ 9, 437–444. 10.2174/1874325001509010437 26587059 PMC4655850

[B31] Stahl S. Vida D. Meisner C. Lotter O. Rothenberger J. Schaller H.-E. (2013). Systematic Review and Meta-Analysis on the Work-Related Cause of de Quervain Tenosynovitis: a Critical Appraisal of Its Recognition as an Occupational Disease. Plastic Reconstr. Surg. 132, 1479–1491. 10.1097/01.prs.0000434409.32594.1b 24005369

[B32] Sun X. Shen Y. Zhou Q. Jia Y. Qiu Z. Li S. (2019). Comparison between acupotomy and local steroid injection for the management of de Quervain disease: a systematic review protocol. Medicine 98, e17765. 10.1097/MD.0000000000017765 31725617 PMC6867724

[B33] Sun G. Li X. Dai J. (2024). The role of personalized stenosing tenosynovitis brace therapy based on 3D printing technology: a prospective cohort study. Health Sci. Rep. 7, e70156. 10.1002/hsr2.70156 39558934 PMC11570869

[B34] Suwannaphisit S. Chuaychoosakoon C. (2022). Effectiveness of surgical interventions for treating de Quervain’s disease: a systematic review and meta-analysis. Ann. Med. and Surg. 77, 103620. 10.1016/j.amsu.2022.103620 35638053 PMC9142670

[B35] Thomson C. E. Crawford F. Murray G. D. (2005). The effectiveness of extra corporeal shock wave therapy for plantar heel pain: a systematic review and meta-analysis. BMC Musculoskelet. Disord. 6, 19. 10.1186/1471-2474-6-19 15847689 PMC1097736

[B36] Vavken P. Holinka J. Rompe J. D. Dorotka R. (2009). Focused extracorporeal shock wave therapy in calcifying tendinitis of the shoulder: a meta-analysis. Sports Health A Multidiscip. Approach 1, 137–144. 10.1177/1941738108331197 23015865 PMC3445068

[B37] Vuillemin V. Guerini H. Bard H. Morvan G. (2012). Stenosing tenosynovitis. J. Ultrasound 15, 20–28. 10.1016/j.jus.2012.02.002 23396894 PMC3558240

[B38] Wei L. Tong Q. Liu Y. Hou X. Zhi F. (2022). Different acupotomy for stenosing tenosynovitis: a protocol for systematic review and network meta-analysis. Medicine 101, e28050. 10.1097/MD.0000000000028050 35029874 PMC8735810

[B39] Wu Z. Wang Y. Ye X. Chen Z. Zhou R. Ye Z. (2021). Myofascial release for chronic low back pain: a systematic review and meta-analysis. Front. Med. 8, 697986. 10.3389/fmed.2021.697986 34395477 PMC8355621

[B40] Xiong Y. Wen T. Jin S. Lin L. Shao Q. Peng Y. (2024). Efficacy and safety of extracorporeal shock wave therapy for upper limb tendonitis: a systematic review and meta-analysis of randomized controlled trials. Front. Med. 11, 1394268. 10.3389/fmed.2024.1394268 39139789 PMC11319137

[B41] Xue D.-P. Yang Z. Liu R. (2016). Clinical efficacy of extracorporeal shock wave therapy for stenosing tenosynovitis of the styloid process of the radius. J. Inn. Mong. Med. Univ. 6, 556–559.

[B42] Yildirim P. Gultekin A. Yildirim A. Karahan A. Y. Tok F. (2016). Extracorporeal shock wave therapy *versus* corticosteroid injection in the treatment of trigger finger: a randomized controlled study. J. Hand Surg. Eur. Vol. 41, 977–983. 10.1177/1753193415622733 26763271

